# A practical examination of RNA isolation methods for European pear (*Pyrus communis*)

**DOI:** 10.1186/s13104-017-2564-2

**Published:** 2017-06-29

**Authors:** Loren Honaas, Elena Kahn

**Affiliations:** US Department of Agriculture, Agricultural Research Service, Physiology and Pathology of Tree Fruits Research Unit, Wenatchee, WA 98801 USA

**Keywords:** RNA, *Pyrus communis*, 2-mercaptoethanol, Dithiothreitol, Cetyl-trimethyl-ammonium-bromide, Bioanalyzer, RNA integrity number, ‘d’Anjou’, ‘Bartlett’

## Abstract

**Objective:**

With the goal of identifying fast, reliable, and broadly applicable RNA isolation methods in European pear fruit for downstream transcriptome analysis, we evaluated several commercially available kit-based RNA isolation methods, plus our modified version of a published cetyl trimethyl ammonium bromide (CTAB)-based method.

**Results:**

We confirmed previous work indicating chaotropic agent-based kits produced sufficient, high-quality RNA in freshly harvested, mature ‘Bartlett’ fruit. However, RNA isolation from ‘d’Anjou’ pear peel and especially cortical tissues of fruit stored for 11 months proved challenging to all but our modified CTAB-based method. Generally, more RNA was recovered from peel tissues than cortical tissues. Less toxic dithiothreitol was confirmed to be an acceptable reducing agent as its substitution for 2-mercaptoethanol often yielded high quality RNA. Finally, we present evidence that erroneous signals in the 5S region of Bioanalyzer RNA size plot histograms, that interfered with RNA integrity number calculation, were small RNA fragments that are reduced by simple cleanup procedures, not artifacts as previously reported.

**Electronic supplementary material:**

The online version of this article (doi:10.1186/s13104-017-2564-2) contains supplementary material, which is available to authorized users.

## Introduction

Obtaining sufficient amounts of high quality RNA from plant tissues is a primary hurdle to studying the transcriptomes of plants. Many plant tissues are recalcitrant to RNA extraction for a host of reasons including abundant secondary metabolites and polysaccharides, tough cell walls that may be lignified, high levels of native RNase activity, and widely variable amounts of RNA [1]. Furthermore, readily available and convenient RNA kits are often only evaluated on a few model plants, leaving evaluation of these products to the researcher. Recent surveys of RNA extraction methods [2–4] across many plant species have typically used primarily young leaves, as these are among the least recalcitrant and easiest to obtain plant tissues.

Climacteric fruits of woody trees, including pear and apple fruit, present challenges to RNA isolation like those mentioned above, but especially due to increases in interfering substances that accumulate during ripening and result in low yields of RNA due to co-precipitation with these substances [5–8]. This issue is especially common when using lysis buffers that contain chaotropic agents [4, 9] such as those found broadly in commercially available kits. However, high quality transcriptome data that followed successful RNA isolation using the Qiagen RNeasy Plant Kit™ [10] has been reported for pear (*Pyrus communis* ‘Bartlett’). This study included developing fruit up to harvest maturity that had entered the ethylene climacteric, but did not include older fruit.

For postharvest fruit physiology, studying fruit pre-harvest through extended storage is routine [11–15], yet surveys of the efficacy of various RNA extraction methods for mature fruit that has been stored, especially for European pear (*P. communis—*e.g. ‘d’Anjou’, ‘Bartlett’) have not been reported. To facilitate efforts for functional genomics of tree fruit towards improving postharvest fruit quality [16], we present here a practical examination of pear fruit RNA isolation methods including several commercially available kits as well as a rapid and robust CTAB-based method. We emphasize obtaining sufficient high quality RNA from small amounts of stored and freshly harvested fruit tissue for sensitive transcriptome analysis applications, including quantitative real time PCR (qPCR) and transcriptome sequencing technologies, in the context of postharvest fruit physiology.

## Main text

### Materials and methods

#### Plant material

‘d’Anjou’ pears (*Pyrus communis)* were obtained from Cashmere, WA on August 31 2015, stored at 33 °F in air for 11 months, and are indicated as “stored”. ‘d’Anjou’ and ‘Bartlett’ pear (also *P. communis*) were obtained from an experimental orchard located near Orondo, WA on August 4 2016 (approximately commercial maturity for ‘Bartlett’ and approximately 2 weeks before commercial maturity for ‘d’Anjou’) and were indicated as “fresh”. ‘Gala’ apples were obtained at commercial maturity August 15 2016 from a commercial orchard located near Mattawa, WA.

#### Commercially available kits tested

We obtained commercially available kits (see list in Table 1) for testing. For every kit we substituted dithiothreitol (DTT, VWR product # 0281-5 g) for 2-mercaptoethanol (B-ME) based upon findings by Mommaerts et al. [17] that the less toxic DTT was a suitable reducing agent during tissue lysis. The manufacturer’s instructions were followed for all kits, including recommended elution volumes. Kit names and catalog numbers are as follows: Macherey–Nagel NucleoSpin^®^ RNA Plant 740949, Omega E.Z.N.A.^®^ Total RNA Kit I R6834-00, Omega E.Z.N.A.^®^ HP Total RNA Kit R6812-00, Omega E.Z.N.A.^®^ Plant RNA Kit R6827-00, Qiagen RNeasy^®^ Plant Mini Kit 74903, Qiagen RNeasy^®^ Plus Universal Kit 73404, Thermo Scientific GeneJET Plant RNA Purification Mini Kit K0809, Zymo ZR Plant RNA MiniPrep™ R2024.

**Table 1:** The kits tested plus the modified CTAB method are listed along with a summary of results

Method	Tissue							
	d’Anjou peel stored	d’Anjou cortex stored	d’Anjou peel fresh	d’Anjou cortex fresh	Bartlett peel fresh	Bartlett cortex fresh	Gala peel fresh	Gala cortex fresh
Macherey–Nagel NucleoSpin RNA Plant buffer RAI Cat # 740949	X	X	–	–	–	–	–	–
Macherey–Nagel NucleoSpin RNA Plant buffer RAP Cat # 740949	√_**a**_	OK_c_	√_**e**_	√_**g**_	√	√	√	√
Omega E.Z.N.A. HP Total RNA Cat # R6812-00	√	X	–	–	–	–	–	–
Omega E.Z.N.A. Total RNA Cat # R6834-00	√	X	–	–	–	–	–	–
Omega E.Z.N.A. Plant RNA Cat # R6827-00	X	X^nr^	–	–	–	–	–	–
ThermoFisher GeneJET Plant RNA Cat # K0809	OK	X	–	–	–	–	–	–
Qiagen RNeasy Plus Universal Cat # 73404	X	Poor	–	–	√	√	–	–
Qiagen RNeasy Plant buffer RLC Cat # 74903	√	Poor	–	–	√*	√*	–	–
Qiagen RNeasy Plant buffer RLT Cat # 74903	OK	X	–	–	–	–	–	–
Zymo ZR Plant RNA Cat # R2024	X	X	–	–	–	–	–	–
CTAB modified from Gapper et al. [18]	√_**b**_	√_d_	√_f_	√_**h**_	√	√	√	√

Pear fruit that was stored for 11 months is indicated as “stored” and freshly harvested pear and apple fruit is indicated as “fresh”.** √** = RIN ≥7.5, yield per ~100 mg ≥100 ng, OK = RIN ≥7.5 (or with clear 28s and 18s peaks) and yield 10–100 ng, poor = RIN 3–7.5 (or with clear 28s and 18s peaks) and/or yield <10 ng, X = RIN <3 and/or yield below detectable limit, – not tested

**Fig. 1:**
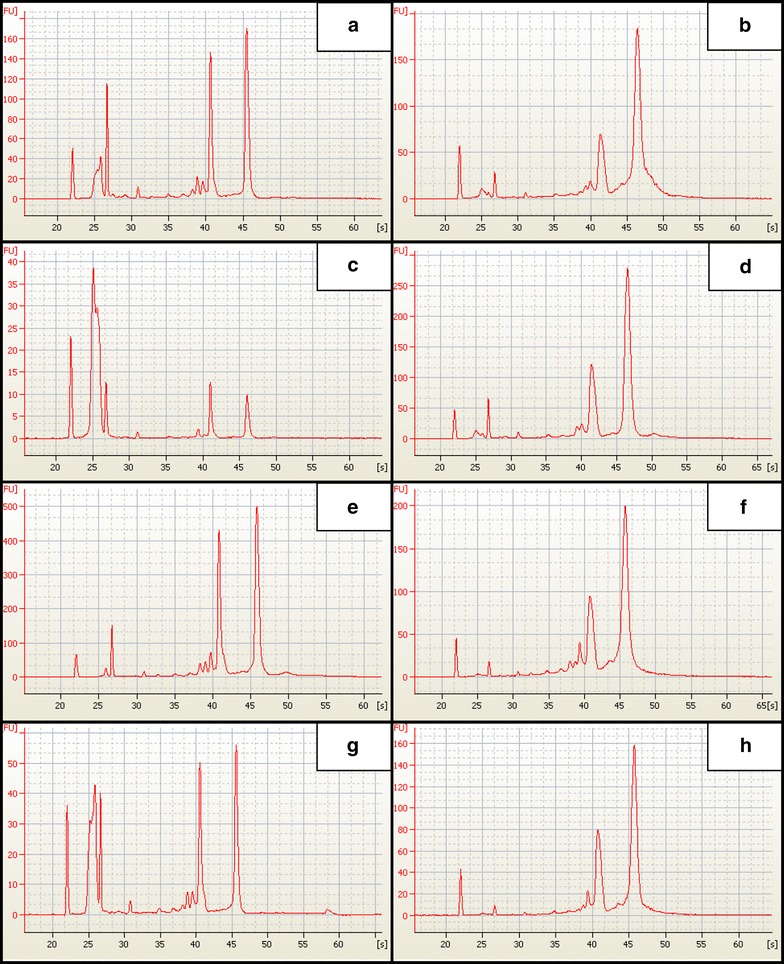
Kit based isolations and CTAB isolations yield intact RNA. Representative RNA profiles of similar tissues. The Y axis is arbitrary fluorescence units (the Agilent Bioanalyzer uses an internal standard to calibrate fluorescence for each run) and the X axis is time. **a**, **b** d’Anjou peel stored, **c**, **d** d’Anjou cortex stored, **e**, **f** d’Anjou peel fresh, **g**, **h** d’Anjou cortex fresh from 2 isolation methods. **a**, **c**, **e**, **g** Macherey–Nagel Nucleospin Plant Kit and the modified CTAB protocol **b**, **d**, **f**, **h**. These plots are referenced by subscript in Table 1

Subscript letter indicates panel in Fig. 1

*nr* not replicated

* Independently validated repeat of published result [10]

#### Modified CTAB

See Additional file 1 for the detailed CTAB protocol. This protocol was modified from Gapper et al. [18] with the following changes: (1) DTT was substituted for B-ME, (2) the lysis (600 µL) and organic phase extraction (chloroform 530 µL) volumes were reduced to accommodate commonly available 1.7 mL microcentrifuge tubes, (3) the organic phase extraction was centrifuged at 12,000×*g* for 15 min at 4 °C, (4) we added an air drying step to enhance ethanol removal and (5) allowed the elution to incubate at RT for 1 min before centrifugation. Diethylpyrocarbonate (DEPC) treated water was used to make solutions used in this protocol and was prepared as described in Sambrook et al. [19].

#### Tissue processing

Fruits were washed with mild dish detergent and water, then rinsed with deionized water. Fruit was peeled with a standard vegetable peeler and peel tissues were flash frozen in liquid nitrogen. Immediately following peeling, the pear was cut in half, and roughly 1 cm sub-epidermal cortical tissue from the equatorial region of the fruit was minced finely and flash frozen in liquid nitrogen. Frozen tissues were ground to a fine powder using a clean mortar and pestle chilled with liquid nitrogen. Frozen tissue powder was transferred to a pre-cooled specimen container using a clean, pre-cooled spatula. The specimen containers were immediately stored at −80 °C. To avoid the tedious and inherently inaccurate estimation of small frozen tissue masses we weighed 100 mg of frozen tissue, and thereafter visually estimated an equivalent tissue mass for each preparation in the interest of time.

#### RNA cleanup

RNA preparations from freshly harvested (“fresh”) d’Anjou peel and cortex using the Macherey–Nagel NucleoSpin Plant RNA Kit and our modified CTAB protocol were pooled and processed with Zymo’s RNA Clean & Concentrator™-5 (cat# R1015) following steps to exclude small RNA fragments (<200 nt) and adhering to the manufactures instructions.

#### Sample analysis

RNA was evaluated with a NanoDrop 2000 spectrophotometer (Thermo Fisher Scientific—http://www.nanodrop.com) to provide sample concentration, A_260/280_ and A_260/230_. The Agilent Bioanalyzer (Agilent Technologies—http://www.agilent.com), using the RNA Pico assay (cat#: 5067-1513) and following the manufacturers’ instructions, provided sample concentration, RNA integrity number (RIN), and other detailed quality information.

#### Data analysis

**Fig. 2:**
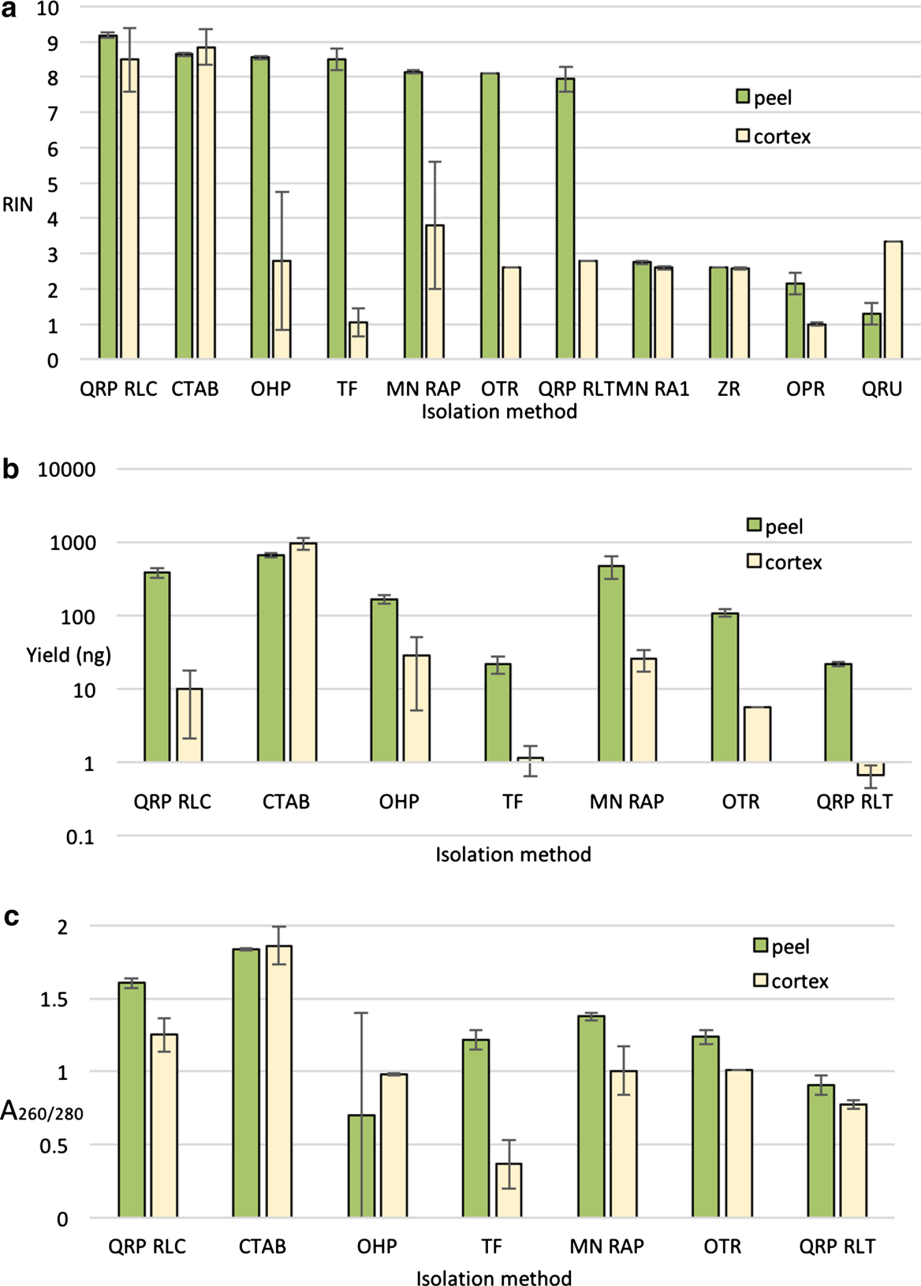
Both yield and quality are variable within and across kit based methods, yet the modified CTAB protocol produces consistent high yield and quality in stored ‘d’Anjou tissues. **a** RINs are higher and more consistent across methods for stored ‘d’Anjou’ peel than cortex. **b** Excluding protocols with degraded RNA, yields are variable across kits with the highest yield using the CTAB protocol. **c** Excluding protocols with degraded RNA, A_260/280−_ ratios were also variable across methods, with CTAB again producing the cleanest RNA. *Error bars* are standard error of the mean, where applicable. Some data are missing due to very low yield or severely degraded individual samples. *QRP RLC* Qiagen RNeasy Plant using buffer RLC, *CTAB* our modified CTAB protocol see Additional file 1, *OHP* Omega EZNA HP total RNA, *TF* thermo fisher, *MN RAP* Macherey–Nagel NucleoSpin Plant using buffer RAP, *OTR* Omega EZNA total RNA, *QRP RLT* Qiagen RNeasy Plant using buffer RLT, *MN RA1* Macherey–Nagel NucleoSpin Plant using buffer RA1, *ZR* ZR plant RNA MiniPrep, *OPR* Omega EZNA plant RNA Kit 1, *QRU* Qiagen RNeasy plus universal

Many samples were at or below the limit of detection for the NanoDrop 2000, thus these data were excluded from our analysis. Samples that had intact 18s and 28s RNA, but had strong erroneous signals in the 5s region (broad peaks of partially degraded and/or small RNAs—see “Results and discussion” and Additional file 3) often produced errors in the Bioanalyzer’s RIN estimation. We manually adjusted the “5s Region Anomaly Threshold” in the Agilent 2100 Expert software (version B.02.07) from 0.5 to 1.0 to override the error and produce a RIN value. Bioanalyzer profiles were examined for other anomalies like bubbles (huge sharp peaks) or blank wells (presumably blocked capillaries) and rerun as necessary. Sample data were recorded in a custom database—an abbreviated version can be found in Additional file 2. Error bars on plots in Fig. 2 are standard error of the mean.

### Results and discussion

The sampling strategy was to evaluate the relatively recalcitrant tissues first (stored d’Anjou pear fruit), followed by less recalcitrant tissues (freshly harvested mature pear and apple fruit). We included apple fruit because the Gapper et al. [18] protocol, on which ours was based, was used successfully on apples and we wished to confirm our modified protocol was useful for apple tissues as well. Based upon the results of the tests in stored ‘d’Anjou’ fruit, the Macherey–Nagel Nucleospin Plant kit was selected for additional testing based on the observation that the low yielding preparations were more or less of equivalent quality to the next best Qiagen RNeasy Plant kit, yet yields were more consistent and higher with the Macherey–Nagel product for stored ‘d’Anjou’ cortical tissues. In addition to the best kit, we tested additional tissues with our modified CTAB method. We selected these two methods because in practice, use of a single method for a range of tissues reduces confounding factors, especially in the context of postharvest fruit physiology where fruit is often stored for many months and evaluated at multiple time points spanning the storage period [11–15].

Ribosomal RNA integrity (frequently estimated by the RNA integrity number (RIN) [20]) has been shown to be an excellent proxy for the integrity of other RNA species present in total RNA preparations, namely mRNA [21]. Furthermore, compared to estimates using samples of equivalent high RNA integrity, relative gene expression measurements estimated with qPCR were shown to become more divergent as RNA integrity decreased, though Imbeaud et al. did find that interrogating similarly degraded RNAs was a possible workaround for dealing with partially degraded RNA samples [21]. It has also been shown that successful transcriptome analysis by second generation sequencing was correlated with RNA integrity and A_260/280_ values [2, 22]. We therefore relied on these metrics to predict success in downstream applications that target mRNA.

Our modified CTAB protocol routinely produced excellent quality RNA with RINs ≥8.3, A_260/280_ ≥1.67 and the best yields across all tissue types ranging from 0.5 to 2 µg from ~100 mg fresh weight tissue (Table 1; Figs. 1, 2). The commercially available kits produced widely variable results, though some were comparable to our benchmark CTAB method. Generally, peel tissues yielded more and better quality RNA than cortical tissues, and younger tissues than older, stored ones. This is not surprising because fruit cortical tissues are specialized for storage and, by mass, contain less RNA compared to peel. Generally older tissues are more recalcitrant to RNA extraction than younger ones for a variety of reasons including presence of co-extracted secondary metabolites [1] and in the case of climacteric fruit, increases in substances that tend to co-precipitate with RNA [5–8]. Kits with alternate buffers, such as the Macherey–Nagel NucleoSpin Plant and Qiagen RNeasy Plant kits, tended to produce better results using the alternate buffers (Table 1), which make them attractive options compared to kits with no alternates.

Importantly, our CTAB based protocol was time equivalent to the kits that were tested, though more equipment intensive (e.g. requiring a fume hood for chloroform handling, a refrigerated centrifuge and a water bath). We eliminated intensive preparation of spatulas and mortars and pestles by baking at 200 °C prior to use because native plant RNase activity [23] renders these steps redundant due to the processing of tissues with liquid N_2_ and the strong denaturing conditions of lysis buffers. While we did replace the reducing agent B-ME with the less toxic DTT, verified as a suitable B-ME replacement by Mommaerts et al. [17], the CTAB method still included more hazardous material compared to most kits tested, excluding the Qiagen RNeasy Plus Universal kit which includes the QiaZol reagent and an organic extraction. However, if yield is not a critical issue (e.g. cDNA synthesis for PCR amplification) or amplification and cleanup of pooled samples is part of a design, then the convenient Macherey–Nagel NuleoSpin Plant or Qiagen RNeasy Plant kit are attractive choices. The CTAB method is more scalable, though given input requirements for modern applications such as transcriptome sequencing (i.e. Illumina TruSeq Stranded mRNA—http://www.illumina.com), preparations yielding as little as 25 ng of total RNA may be sufficient. This is especially relevant when the target tissues are very small or hard to generate because a brute force approach of processing large amounts of tissue may not be practical or even possible.

Estimations of RNA integrity, as determined by the RIN (estimated during Bioanalyzer analysis), were hampered by what we hypothesized were small fragments of RNA (Fig. 2, C&G; Additional file 3). Jordon-Thaden and Chanderbali [2] speculated that broad peaks in the 5s region of the RNA histogram (Additional file 3) were due to Bioanalyzer marker contamination. Yet the kit chemistry has been rigorously tested and the dyes are highly specific to RNA (http://www.chem-agilent.com—Publication Number 5988-7650EN), making erroneous non-RNA signals unlikely. To test this hypothesis, we chose 8 samples (4 pairs) to clean-up with Zymo’s RNA Clean & Concentrator-5, which is a kit that includes a step to selectively remove small RNAs (<200 nt). Use of the clean-up process resulted in a substantial reduction in the magnitude of the broad peak seen in our RNA preps in the 5s region (Additional file 3), supporting the hypothesis that this erroneous signal was due, at least in part, to small RNA fragments. These fragments may include intact small RNAs as well as partially degraded RNAs. The erroneous 5s region signal seemed to interfere with RIN estimation especially for samples at low concentrations (Additional file 3). It may be possible to remove these fragments with a similar cleanup scheme for the low yielding kits tested here, but recovery of extremely low abundance samples following cleanup may be difficult. Our CTAB method did routinely produce some weak small RNA fragment signals in the 5s region, but it was substantially less than the kit-based methods (Fig. 1).

Based on the results of our comparison, it is clear that our short and reliable modified CTAB protocol would be an excellent choice for European pear fruit at or near harvest maturity through to fruit that had been stored for an extended time. The Macherey–Nagel NucleoSpin Kit and Qiagen RNeasy Plant kit represent the best of the kits we tested—in part because of available alternate lysis buffer chemistry. The kits are less robust than our CTAB method, but with affordable and easy kit based cleanup (e.g. Zymo RNA Clean & Concentrator), the hurdles that low yields and partially degraded RNA pose could be largely overcome. The input amount for all methods tested here was around 100 mg, though when processed with a successful method, the plant tissue yielded 500 ng–2 µg of high quality clean RNA, which is sufficient for a range of state-of-the-art transcriptome analysis methods.

## Additional files

**Additional file 1.** Detailed protocol for our modified CTAB method.

**Additional file 2.** Abbreviated database for all isolations done in this study.

**Additional file 3.** Signal in the 5s region is likely due to small RNA fragments and can interfere with RIN estimation. A, B are replicates of RNA isolations from cortical fruit tissue of freshly harvested d’Anjou pear. C—samples shown in A, B were pooled, and processed with Zymo’s RNA Clean & Concentrator Kit-5 in which we opted to remove RNA fragments <200 nt. The arrows indicate the putative small RNA fragments that are reduced during the cleanup (which is designed to remove small RNA fragments). D—low yield RNA prep with clear 18s and 28s peaks and low signal in the 5s region produces a RIN of 8.5, E—similarly low yielding prep of RNA with clear 18S and 28S peaks but with high signal in the 5S region produces a RIN of 2.8. The portion of intact mRNA in these preps likely does not differ substantially.

## Abbreviations

CTAB: cetyl trimethyl ammonium bromide
DTT: dithiothreitol
RIN: RNA integrity number
