# Supplementary material for: A practical examination of RNA isolation methods for European pear (Pyrus communis)
Source: BMC Res Notes. 2017 Jun 29;10:237. doi: 10.1186/s13104-017-2564-2 (PMC5492931; doi:10.1186/s13104-017-2564-2)
Supplement: Supplementary file 1 — Additional file 1. Detailed protocol for our modified CTAB method. [file 13104_2017_2564_MOESM1_ESM.docx]

**Supplement 1**

**Modified CTAB protocol for pear and apple fruit**

This protocol was modified from the method used in Gapper et al. (2013).

**General Procedural notes:**

*Workspace preparation-*

To avoid RNA degradation by RNase contamination, the benchtop, pipettes, centrifuges, tube racks, and any surfaces on which RNA work would be performed were wiped down thoroughly with 70% ethanol. All disposable plastics (microcentrifuge tubes, sterile filter pipette tips) were certified RNase free. All buffers (and plastics that were not prepackaged) were prepared/stored in containers that were treated with RNaseZAP, rinsed with sterile diethlypyrocarbonate (DEPC-1%) treated water, and air dried.

*Tissue homogenization with liquid N_2_ -*

Pear and apple fruit were washed with mild dish detergent and water, then rinsed with DI water. Fresh tissue was flash frozen in liquid N_2_-filled specimen containers (VWR cat#25384-148) that were floating in a liquid nitrogen bath. A small amount of frozen tissue was transferred to a chilled mortar (approx. 65mm ID), where they were finely ground with a chilled pestle. Frozen tissue powder was transferred to a pre-chilled specimen container using a clean, pre-chilled metal spatula. The specimen containers were immediately stored at -80°C.

**Reagents:**

**Buffer:**  **For 250 mL**

- 2% Cetyl Trimethyl Ammonium Bromide (CTAB) 5 g
- 2% Polyvinylpyrrolidone (PVP, MW av. 40,000) 5 g
- 100 mM Tris HCl pH 8.0 25 mL (1 M)
- 25 mM EDTA pH 8.0 10 mL (0.5 M)
- 2M NaCl 100 mL (5 M)
- 0.5 g/L spermidine 0.125 g
- *40 mM Dithiothreitol (DTT, added just before use) 20 µL 2 M stock per 1 mL lysis buffer

Bring buffer to 250 mL with DEPC treated water. Store at room temperature.

*Add 20 µL 2M DTT per 1 mL CTAB lysis buffer. Place CTAB buffer aliquot + DTT in 15 mL conical tube and equilibrate to 70°C in a water bath before use.

**Other reagents/chemicals**

100% ethanol

80% ethanol (in conical tube- for wash steps)

70% ethanol (in spray bottle for cleaning)

Chloroform (store in glass bottle!)

Liquid nitrogen

RNase-free water

RNaseZAP (Sigma)

**Materials:**

Foam tube floats (for incubating 1.7 mL tubes with tissue + CTAB buffer in water bath)

Micropipettes and sterile, RNase free-barrier tips

1.7 mL microcentrifuge tubes RNase free

Epoch spin columns (catalog #1940-050)

Paper towels

Kimwipes

**Equipment:**

Refrigerated benchtop microcentrifuge (4°C)

Benchtop microcentrifuge

Fume hood

Vortexer

Water bath (70°C)

Heat block (70°C)

Ice bucket

-20˚C & -80˚C freezer

Liquid nitrogen Dewar

**Procedure:**

**Notes prior to starting:**  Set water bath and heat block to 70°C; cool centrifuge down to 4°C. Wear gloves and proper PPE (goggles, lab coat). Clean workspace and pre-label 1.7 mL tubes. Prepare CTAB lysis buffer aliquot in a 15 mL conical tube. (e.g. 8 mL CTAB plus 160 µL 2 M DTT for 4 pairs of isolations). Warm the aliquot in a 70°C water bath. Fill ice bucket for eluted RNA samples, and obtain the frozen tissue from -80°C freezer (transport using small Dewar with liquid nitrogen).

1. **Lyse 100 mg frozen, ground tissue in 600 µL of 70°C lysis buffer, vortex immediately.**
   1. Aliquot 600 µL lysis buffer into an RNase-free 1.7 mL tube and place in 70°C water bath. Add tissue to warm tube + buffer and vortex immediately.

**NOTE:** Tubes containing warm lysis buffer can be placed in a 70°C heat block on the bench, so tissue transfer is more convenient.

1. **Incubate at 70^o^C for 10 min with vigorous shaking/vortexing.**
   1. Vortex 2-3 times during incubation and before adding chloroform.

**NOTE:** Keep tubes in foam floats in water bath during incubation period, except to vortex. Blot tubes dry before vortexing.

1. **Add 530 µL Chloroform (in fume hood), vortex 10 sec, then spin at 12000 x g at 4°C in pre-cooled centrifuge for 15 min.**

**NOTE:** Spinning at RT at top speed (21000 x g) for 10 minutes caused tube failure. We reduced the speed and temperature to improve tube integrity and increased spin time to compensate for reduced g-force.

1. **Carefully remove the aqueous layer and transfer to a new RNase-free 1.7 mL tube.**
   1. It may be preferable to remove the aqueous layer in a few steps using a smaller volume pipette. Total aqueous phase volume is ~550 µL. To avoid contamination, transfer 500 µL leaving a small amount of the aqueous phase. This also simplifies the estimation of ethanol to add for the precipitation (250 µL).
2. **Add 0.5 volumes 100% ethanol (usually ~250 µL); mix by pipetting 10X. Without delay, transfer to an Epoch RNA column. Spin at 8000 x g for 30 sec; discard flow-through.**
3. **Wash with 500 µL 80% ethanol followed by a spin @ 8000 x g for 30 sec; discard flow-through.**
4. **Repeat wash - 500 µL 80% ethanol followed by a spin @ 8000 x g for 30 sec; discard flow-through.**
5. **Spin column for 2 min at top speed (e.g. 21000 x g) to remove ethanol before elution.**
6. **Elute RNA in 50 µL RNase-free H_2_O and then spin at 8000 x g for 1 min.**
   1. Place columns in fresh 1.7 mL tubed with open columns and let air dry for 1 minute before eluting to remove trace ethanol. Add water (or elution buffer), then place in centrifuge and wait 1 minute to start the spin. Place eluted RNA immediately on ice, store short term at -20°C or long term at -80˚C.
