# Supplementary figures and images for: A practical examination of RNA isolation methods for European pear (Pyrus communis)
Source: BMC Res Notes. 2017 Jun 29;10:237. doi: 10.1186/s13104-017-2564-2 (PMC5492931; doi:10.1186/s13104-017-2564-2)

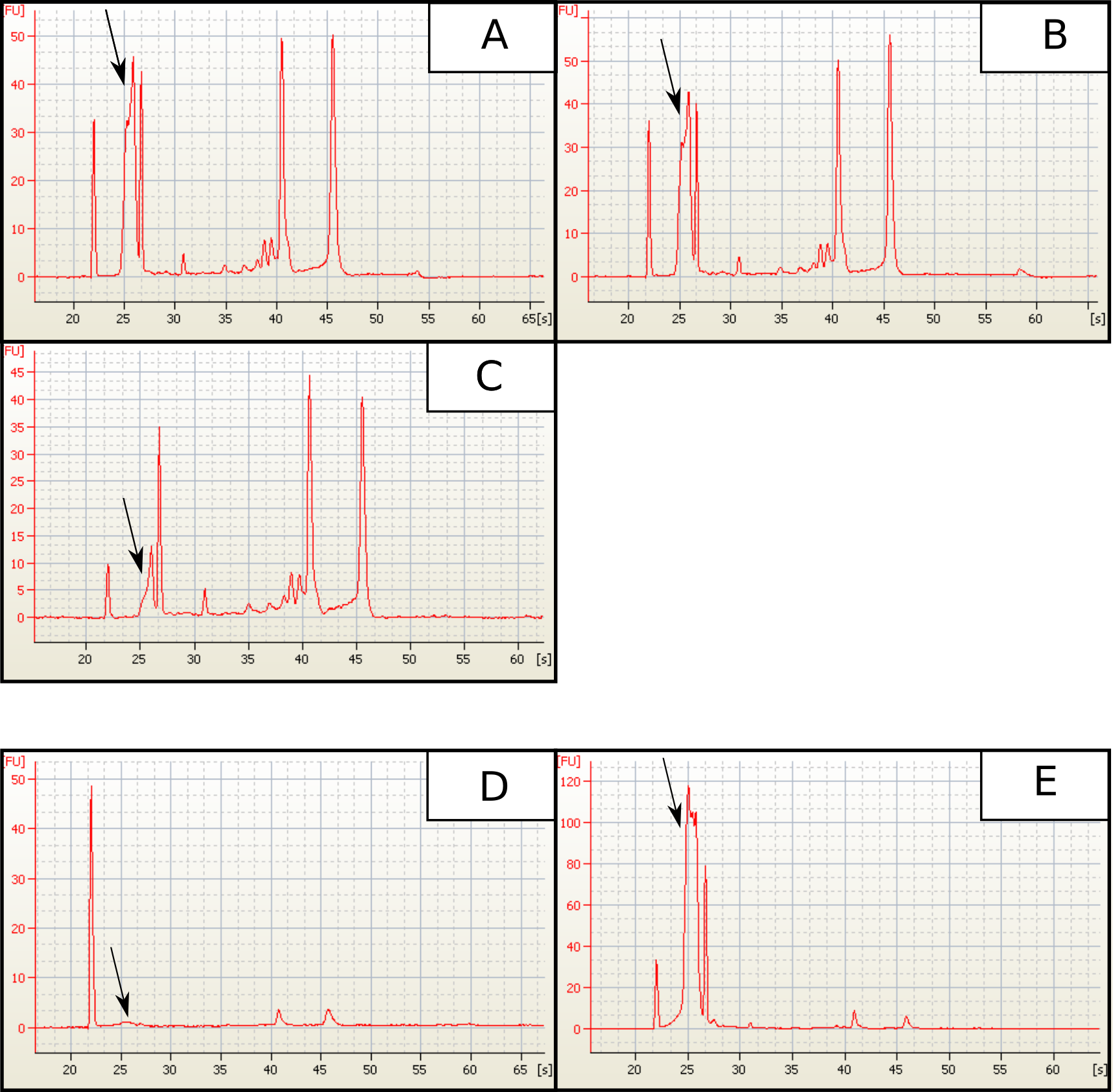

Supplement: Supplementary file 3 — Additional file 3. Signal in the 5s region is likely due to small RNA fragments and can interfere with RIN estimation. A, B are replicates of RNA isolations from cortical fruit tissue of freshly harvested d’Anjou pear. C—samples shown in A, B were pooled, and processed with Zymo’s RNA Clean & Concentrator Kit-5 in which we opted to remove RNA fragments <200 nt. The arrows indicate the putative small RNA fragments that are reduced during the cleanup (which is designed to remove small RNA fragments). D—low yield RNA prep with clear 18s and 28s peaks and low signal in the 5s region produces a RIN of 8.5, E—similarly low yielding prep of RNA with clear 18S and 28S peaks but with high signal in the 5S region produces a RIN of 2.8. The portion of intact mRNA in these preps likely does not differ substantially. [file 13104_2017_2564_MOESM3_ESM.png]
